# Two Cases of Immune Checkpoint Inhibitor-Induced Myocarditis With Complete Atrioventricular Block

**DOI:** 10.7759/cureus.36446

**Published:** 2023-03-21

**Authors:** Hisaya Kondo, Jin Kirigaya, Yasushi Matsuzawa, Kiyoshi Hibi

**Affiliations:** 1 Cardiology, Yokohama City University Medical Center, Yokohama, JPN; 2 Emergency and Critical Care, Yokohama City University Medical Center, Yokohama, JPN

**Keywords:** myocarditis, immune-related adverse event, complete atrioventricular block, cardio-oncology, case report

## Abstract

Immune checkpoint inhibitor-induced myocarditis (ICIM) has been reported to be complicated by a complete atrioventricular block. Even though steroids are used in the treatment thereof, there is no standard protocol for their use. We report two cases of ICIM with a complete atrioventricular block with different outcomes. In the first case, the complete atrioventricular block did not recover. In contrast, in the second case, the complete atrioventricular block did recover. We discuss the different courses and outcomes of the two cases in relation to steroid use.

## Introduction

Immune checkpoint inhibitors (ICI) are widely used to treat various cancer types. As more cancer patients are being treated with ICI, the number of immune-related adverse events has increased [1]. The frequency of ICI-induced myocarditis (ICIM) is estimated to be around 0.04% to 1.14%, and the mortality rate has been reported as up to 25% to 50% [2,3]. During hospitalization, 68% of patients with ICIM experience conduction disorders, and complete atrioventricular block (CAVB) occurs in 17% of these patients [4]. Immune checkpoint inhibitor-induced myocarditis with concurrent CAVB is associated with 2.6 times higher mortality rates [4]. However, methods to detect ICIM early and appropriately treat CAVB have not yet been established. Here, we report two cases of ICIM with CAVB with different outcomes. We discuss the possible reasons for the difference in the courses of these two patients.

## Case presentation

Case 1

**Figure 1:**
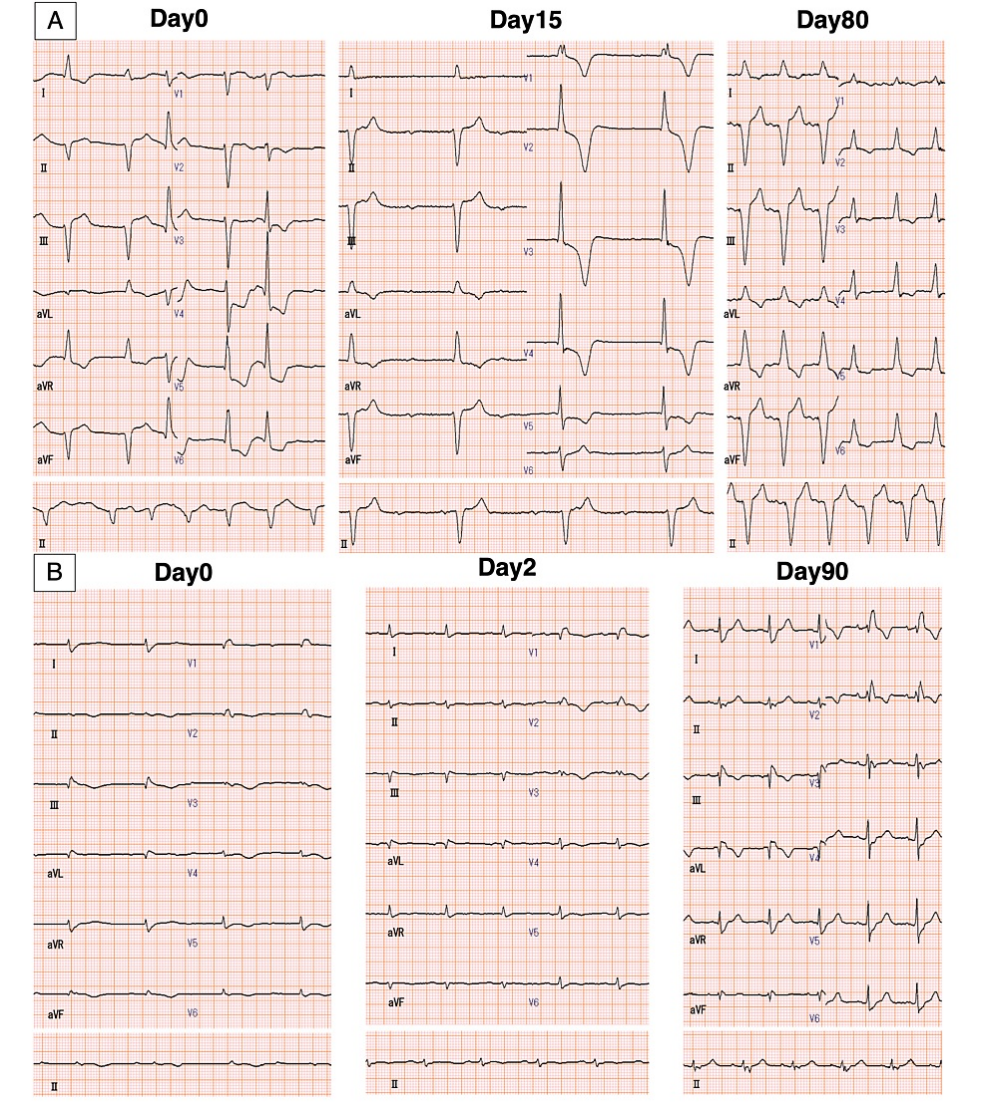
Electrocardiography of the two patients over the course of their admission A: Progressive electrocardiography changes over time in Case 1 with ICIM-complicated myasthenia gravis B: Progressive ECG changes over time in Case 2 with ICIM ICIM: Immune checkpoint inhibitor-induced myocarditis

A 65-year-old man with esophageal cancer and bone metastasis received pembrolizumab after two courses of combination therapy with fluorouracil and cisplatin as first-line therapy and radiation therapy. The patient presented with posterior neck pain, hoarseness that worsened in the evening, and lower-extremity fatigue 50 days after receiving the initial ICI dose. The patient’s vital signs were as follows: respiratory rate 22/min; body temperature 36.8 °C; blood pressure 145/91 mmHg; heart rate 86 bpm; and oxygen saturation 99% on room air. Laboratory tests revealed elevated serum creatine kinase (9405 U/L), troponin I (8.484 ng/ml), and aldolase (196.8 U/L). Electrocardiography (ECG) showed sinus arrest and idioventricular rhythm (Figure 1A).

**Figure 2:**
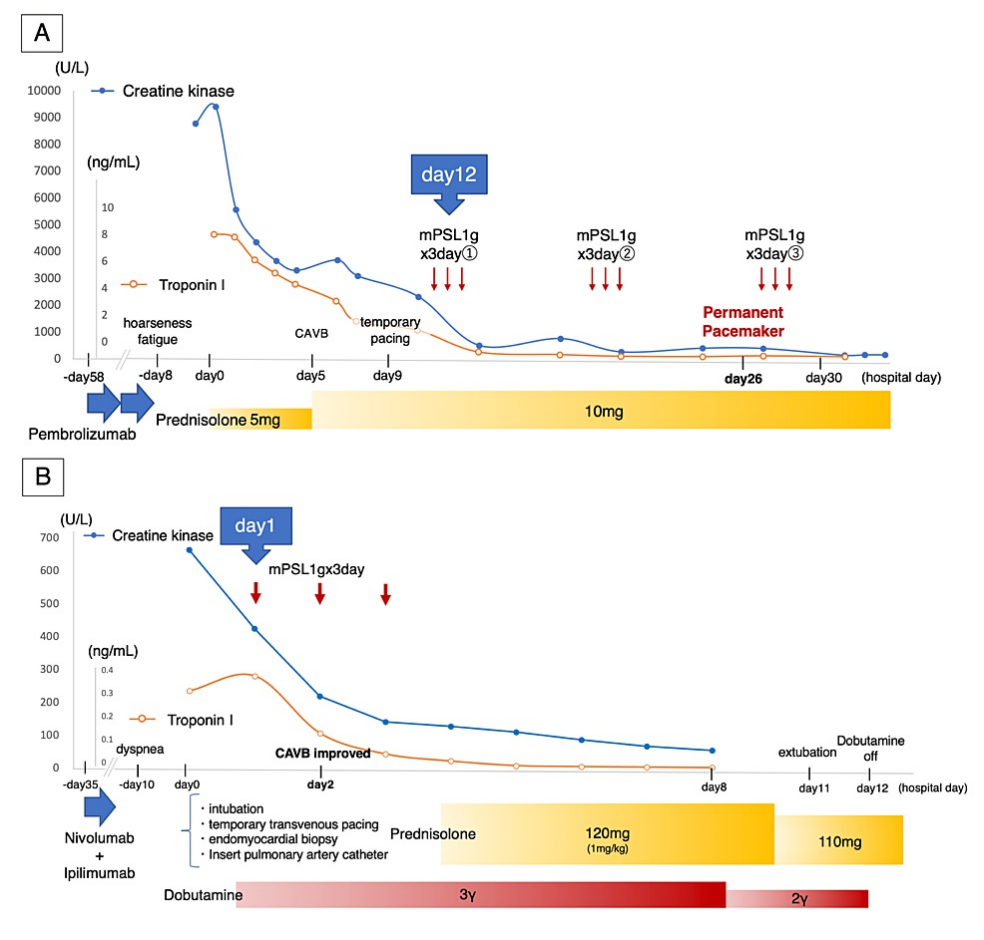
The clinical course of both patients A: The clinical course of Case 1 with ICIM-complicated myasthenia gravis B: The clinical course of Case 2 with ICIM mPSL: Methylprednisolone, CAVB: Complete atrioventricular block, ICIM: Immune checkpoint inhibitor-induced myocarditis

Chest radiography showed no significant congestion or pleural effusion. Transthoracic echocardiography revealed a new-onset mildly depressed left ventricular ejection fraction of 45% to 50%, with neither ventricular hypertrophy nor segmental wall motion abnormalities (see Appendices: videos 1, 2). To examine the patient’s fatigue with diurnal variation, we performed a repetitive nerve stimulation test, which showed a waning pattern in the trapezius muscle. Needle electromyography showed a myogenic disorder in the right biceps muscle, indicating myasthenia gravis and acute myositis. A biopsy of the left biceps muscle revealed lymphocytic infiltration of the myocytes and interstitial space with mild muscle fiber injury, consistent with acute myositis. We clinically diagnosed ICIM with myasthenia gravis and myositis. The patient’s clinical course is shown in Figure 2A. 

First, we discontinued ICI on admission, and due to the risk of transient exacerbation of the myasthenia associated with methylprednisolone (mPSL) pulse therapy, we initiated low-dose prednisolone (5 mg/day). Although the serum creatine kinase and troponin I level steadily decreased, the creatine kinase level increased, and CAVB occurred on day 5. Therefore, we increased the prednisolone dose to 10 mg/day. However, ECG showed no improvement in atrioventricular conduction, and the patient presented with syncope. We inserted a temporary transvenous pacemaker. On day 12, the disease status showed no improvement, and we initiated intravenous mPSL pulse therapy at a dose of 1000 mg/day for three days. However, the atrioventricular conduction defect was irreversible and a permanent pacemaker was implanted. Treatment of the myasthenia required a prolonged hospital stay, and the patient was discharged on day 80. The ECG showed no improvement in atrioventricular conduction (as seen above in Figure 1A). Coronary computed tomographic angiography revealed no significant coronary artery stenosis.

Case 2

A 54-year-old man with malignant nasal melanoma and lymph node metastasis received pembrolizumab and ipilimumab after heavy particle therapy as first-line therapy. The patient presented with dyspnea and leg edema 35 days after the initial ICI dose. The patient’s vital signs were as follows: respiratory rate 24/min, body temperature 36.6 °C, blood pressure 107/78 mmHg, heart rate 71 bpm, and oxygen saturation 100% on room air. Laboratory tests revealed elevated levels of serum creatine kinase (636 U/L), high-sensitivity troponin I (0.307 ng/mL), and creatinine (1.88 mg/dL). Initial ECG revealed a CAVB (Figure 1B), and chest radiography showed congestion and pleural effusion. Transthoracic echocardiography revealed a new-onset, mildly depressed left ventricular ejection fraction of 35% with neither ventricular hypertrophy nor segmental wall motion abnormalities (see Appendices: videos 3, 4). The patient was clinically diagnosed with ICIM. The patient’s clinical course is shown in Figure 2B. 

On admission, considering the low cardiac output syndrome associated with myocarditis, we performed tracheal intubation and inserted a temporary transvenous pacemaker to improve the vital signs. Pulmonary artery catheter measurements revealed a low cardiac index of 0.98 L/min/m2 and a high pulmonary capillary wedge pressure of 26 mmHg. We diagnosed heart failure with a Forrester subset IV and initiated dobutamine at 3 μg/kg/min. The ICIs were discontinued on admission, and intravenous mPSL pulse therapy was initiated within 24 hours of admission at a dose of 1000 mg/day for three days. The troponin I level decreased rapidly, pulmonary artery catheter findings improved, and atrioventricular conduction recovered. Subsequently, the patient was started on 120 mg of prednisolone per day (1 mg/kg/day). The dobutamine level decreased, and the patient was extubated on day 11. We performed a right ventricular endocardial biopsy upon admission, which revealed degeneration of myocardial cells with a high degree of cluster of differentiation (CD)3-dominant lymphocyte infiltration, compatible with myocarditis. The steroid dose was reduced by 10 mg every five days, and the patient was discharged on day 48. The ECG changes over time showed an improvement in atrioventricular conduction (as seen above in Figure 2B). Coronary computed tomographic angiography revealed no significant coronary artery stenosis.

## Discussion

Here, we report two cases of ICIM with CABV with different outcomes. We discuss the possible reasons for the difference in the course of these two patients and how this information can be used for future treatments.

Differences in therapeutic strategy between the two cases

We speculate that the different outcomes of the two cases may be associated with the differences in the therapeutic strategies for ICIM (Table 1).

**Table 1:** Differences between Case 1 and Case 2 mPSL: Methylprednisolone, PSL: Prednisolone

	Case 1	Case 2
Age (in years)	65	54
Sex	Male	Male
Primary disease	Esophageal cancer with bone metastasis	Nasal malignant melanoma with lymph node metastasis
Cancer therapy	Pembrolizumab after fluorouracil and cisplatin for six weeks and radiation therapy	Pembrolizumab and ipilimumab after heavy particle therapy
Time from the cancer therapy initiation to admission	50 days	25 days
Form of onset	Posterior neck pain, hoarseness that worsened in the evening, and lower extremity fatigue	Dyspnea and legs edema
Time from the symptom onset of cardiomyopathy to admission	8 days	10 days
Cardiogenic shock	-	+
Cardiac rhythm	Sinus arrest	Sinus arrest, complete atrioventricular block
Findings of ultrasound examination	Ejection fraction of 45% to 50% with neither ventricular hypertrophy nor segmental wall motion abnormalities	Ejection fraction of 35% with neither ventricular hypertrophy nor segmental wall motion abnormalities
Initial troponin I value (ng/dl)	8.484	0.3
Max troponin I value (ng/dl)	8.484	0.366
Treatment for myocarditis	Days 1: PSL 5 mg/day, day 6: PSL 10 mg/day, day12: 14 mPSL 1000 mg/day, day 19: 21 mPSL 1000 mg/day, day 27: 29 mPSL 1000 mg/day	Days 1-3: mPSL 1000 mg/day, day 4: 9 PSL 120 mg/day reduce the dose by 10 mg every 5 days
Ejection fraction after treatment	45% to 50%	60%
Heart rhythm after treatment	Complete atrioventricular block (pacemaker rhythm)	Sinus rhythm, first-degree atrioventricular heart block, and complete right bundle branch block

The discontinuation of ICI and early initiation of high-dose steroids are essential for ICIM management [5]. However, Case 1 was complicated by myasthenia gravis, a life-threatening immune-related adverse event, which interfered with steroid pulse induction [6,7]. The differences between ICIM- and ICI-induced myasthenia gravis are summarized in Table 2. 

**Table 2:** Difference between ICI-induced myocarditis and ICI-induced myasthenia gravis mPSL: Methylprednisolone, PSL: Prednisolone, ICI: Immune checkpoint inhibitor

	Myocarditis	Myasthenia gravis
Frequency	0.04% to 1.14% [2]	0.12% [7]
Time from the cancer therapy initiation to symptom onset	34 days [2]	29 days [7]
Mortality rate	25% to 50% [3]	28% to 30% [8]
Treatment	Early use of intravenous mPSL 1000 mg/day followed by long-term PSL (1.0–2.0 mg/kg/day) [5]	Initiation of acetyl-cholinesterase inhibitors, and early use of PSL (0.75-1.0 mg/kg/day) [8]
Response rate	50% [5]	63% [8]
mPSL pulse	Use within 24 hours is recommended [5]	Use may cause transient worsening of the symptom in 50% of patients for 2-5 days [7]
Median time to improvement from initiation of steroid	4-6 weeks, steroids can be discontinued [5]	4-5 months, low dose (5 mg) PSL is continued [8]

Both ICIM- and ICI-induced myasthenia gravis are treated with steroids [8]. Early high-dose steroid therapy for ICIM improves the prognosis. Conversely, high-dose steroid therapy for myasthenia gravis may cause transient exacerbation of symptoms, including respiratory failure requiring mechanical ventilation [9]. The mechanisms underlying these side effects remain unclear. Recent hypotheses include the action of antibodies released from degraded lymphocytes, increased cholinesterase activity in neuromuscular junctions, and an overall increase in immune reactions [10]. Owing to the possibility of this serious side effect, we could not initiate early high-dose steroids in Case 1. The mechanism of CAVB complicated by ICIM is also not fully understood and is believed to be modified by various factors. However, our two cases indicate that the early introduction of high-dose steroids might be associated with an improvement in atrioventricular conduction.

There is no consensus on the use of steroids in cases of ICIM complicated with both CAVB and myasthenia gravis. However, in previous case reports of similar cases, some patients were treated with steroid pulse therapy, prioritizing the treatment of myocarditis [11-14]. In these reports, atrioventricular conduction improved after steroid pulse therapy. These findings indicate that in Case 1, intensive care management using early steroid pulse therapy with mechanical ventilation at the time of hospitalization may have improved atrioventricular conduction. Further studies on the dilemma associated with steroid use in cases like these are warranted.

Early detection and therapeutic intervention for ICIM with CAVB

The appropriate dose and timing of steroid administration for ICIM with CAVB in myasthenia gravis remain unclear. However, early detection and therapeutic intervention may improve CAVB complicated by ICIM. In previous case reports, CAVB with ICIM recovered to a normal sinus rhythm with early detection and subsequent immunosuppressive therapy [15]. 

For the early detection of ICIM, the European Society of Cardiology guidelines recommend confirming baseline ECG and echocardiographic information before ICI administration. Additionally, ECG and troponin I measurements after each course, up to the fourth course, are recommended [5]. However, the precise mechanisms underlying ICIM development are unclear, and it is, therefore, difficult to predict. Furthermore, although acute myocarditis is typically associated with chest pain and dyspnea, patients often present with atypical symptoms, such as general fatigue or symptoms of infectious disease [16,17]. Therefore, the European Society of Cardiology monitoring protocol may not be sufficient for the early detection of ICIM. 

We recommend three additional methods for the early detection of CAVB during ICI treatment. First, the patient’s background and baseline ECG findings should be carefully evaluated prior to ICI therapy induction. A population-based cohort study demonstrated that older age, previous myocardial infarction, male sex, suboptimal blood pressure, and fasting glucose levels were associated with the development of atrioventricular block [18]. Baseline ECG findings, such as first-degree atrioventricular block, bundle branch block, and prolonged QRS duration have also been associated with cardiovascular events [19]. These background factors should be considered before initiating ICI therapy. Second, oncologists and cardiologists should collaborate to monitor the ECG results, myocardial enzymes, and echocardiographic findings when patients who received ICI report any of these symptoms or have known risk factors for CAVB. Close, multidisciplinary monitoring of these patients may lead to the early detection of ICIM. Third, since symptoms are scarce in the early stages of the disease and may be preceded only by ECG changes, 24-hour continuous monitoring devices such as smartwatches, may lead to earlier diagnosis and improved outcomes [20]. The establishment of early detection methods may improve patient outcomes.

This report had some limitations. First, in Case 1, we did not perform a myocardial biopsy and ICIM was diagnosed based only on clinical findings. Second, a baseline ECG before ICI initiation was not performed in Case 1, and the possibility that the patient was at high risk of atrioventricular conduction defects cannot be ruled out. 

## Conclusions

An ICIM-associated CAVB is reversible, and early, high-dose steroid administration tends to be effective. Even though this disease is rare, given the possible increased use of ICI therapy in the future, it has been suggested that the occurrence of ICIM may also increase. By accumulating these cases, we can consider the appropriate treatment for ICIM with CAVB.
